# A systematic review of binge drinking interventions and bias assessment among college students and young adults in high-income countries

**DOI:** 10.1017/gmh.2024.24

**Published:** 2024-02-23

**Authors:** Laurencia Bonsu, Priyambda Kumra, Asma Awan, Manoj Sharma

**Affiliations:** 1 Department of Social & Behavioral Health, School of Public Health, University of Nevada, Las Vegas, NV 89119, USA; 2 Department of Environmental and Occupational Health, School of Public Health, University of Nevada, Las Vegas, NV 89119, USA; 3 Department of Internal Medicine, Kirk Kerkorian School of Medicine, UNLV, Las Vegas, NV 89106, USA

**Keywords:** binge drinking, heavy episodic drinking, interventions, college students, young adults, problem drinking

## Abstract

Alcohol is the number one substance used by young people and people of college age. Binge drinking (BD) in this age group is considered one of the most important global health issues, as much harm accrues from it and even lives are lost. This study aimed to review the interventions to curb BD or encourage responsible drinking among college students and young adults. MEDLINE (PubMed), ERIC and APA PsycINFO were searched. The selected articles were published in English and had to evaluate a BD reduction program through a randomized control trial (RCT) among college students or young adults between the ages of 17–24 years. The exclusion criteria included research not published in English, systematic review articles, qualitative studies, designs other than RCTs and discussion articles on college students drinking with no findings. The three reviewers independently screened and extracted the data using the PRISMA guidelines. The overall quality of the studies was assessed. Then, 10 of the 12 interventions studied were found to be successful in reducing BD among college students, though the effect sizes were small to medium. A minority of the studies used behavior change theories. Effective interventions for reducing BD among college students and young adults should include robust behavior change theories, longer follow-up time and the operationalization of multiple outcomes. Process evaluation is needed to be conducted in these studies.

## Graphical abstract

**Figure:**
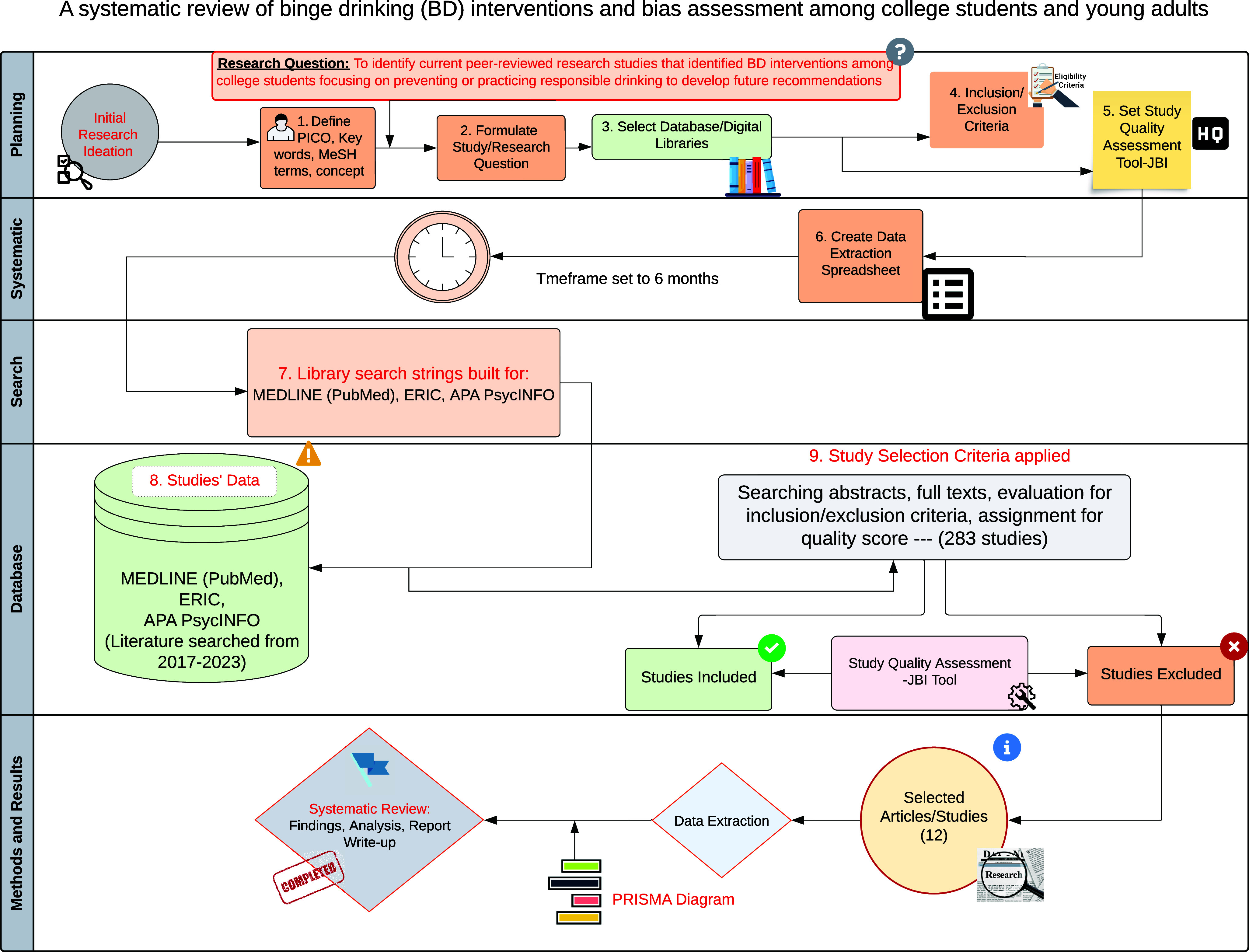

## Impact statement

College and university students are at high risk for binge drinking. Our systematic review focused on preventative interventions directed toward college and university students and young people who may be at increased risk of developing binge drinking behavior and their sequelae. We sought to identify the characteristics of efficacious interventions and develop recommendations. The review has provided evidence that some interventions are being provided to overcome this problem behavior among college students and young adults. However, there is a need to develop more robust interventions based on newer behavior change models. Such interventions would alter the trajectory of binge drinking disorder and its serious health problems, along with the cost associated with binge drinking. This systematic review would lead to valuable clinical and educational research to prevent binge drinking in youth. This review would also pave the way for policy changes for early intervention programs, strategic planning and controlling underage drinking.

## Introduction

Substance abuse and its repercussions affect young adults’ families, communities and society as a whole (Das et al., 2016; Lipari and Van Horn, 2017). Substance use relates to increased morbidity and mortality among youth, with substantial consequences such as missed classes, sexual and physical assault, sexually transmitted infections and even death (DiFulvio et al., 2012; Sharma et al., 2018; Hennessy et al., 2019). The prevalence of alcohol use among young adults under the age of 21 was 30%, based on data from the Substance Abuse and Mental Health Services Administration (SAMHSA) in 2022. The most used substance is alcohol, with one in every eleven young adults reporting binge drinking (BD) (American Addiction Centers, 2023). A blood alcohol concentration of 0.08% or 0.08 g of alcohol per deciliter or greater is the threshold for BD, according to the National Institute on Alcohol Abuse and Alcoholism (NIAAA, 2023). According to the NIAAA (2023), and the SAMHSA (2023), this pattern corresponds to an average adult consuming five or more drinks (for men) or four or more drinks (for women) within 2 h. BD among college students has been shown to have an impact on the social, physical and academic lives of both binge drinkers and non-binge drinkers (NIAAA, 2022; SAMHSA, 2022). According to 2019 countrywide research, over 53% of full-time college students aged 18–22 used alcohol, with approximately 33% BD during the same time period (NIAAA, 2019; SAMHSA, 2019). According to SAMHSA (2021), alcohol contributed to 599,000 unintentional injuries, 97,000 sexual assault cases, including acquaintance rape, suicide attempts, vandalism, 696,000 physical assaults and 3,360,000 driving under the influence. According to an earlier prediction by NIAAA, 43,000 women and 97,000 men were projected to die from alcohol-related causes in 2022. In the United States, BD is the fourth-leading preventable cause of mortality (Bock et al., 2021).

Excessive alcohol use is one of the most significant social medical costs, costing more than $250 billion per year (Sacks et al., 2015; Kazemi et al., 2017). Interest in BD has grown in recent decades, resulting in an increase in the number of scholarly studies, although there is always an opportunity for improvement. A recent nationwide study in the United States found that approximately 24% of 19–20-year-olds had engaged in BD in a 2-week period (Patrick and Terry-McElrath, 2017). BD is also more prevalent among college students than among non-college students, and it increases as young adults enter college due to a lack of parental guidance on campus (NIAAA, 2023; Welsh et al., 2019). In the United States, 38% of 18–22-year-old college students reported indulging in BD in the preceding month, compared to 33% of non-college students (Norman et al., 2019; SAMHSA, 2015). More than 60% of university students in the United Kingdom reported engaging in BD (Norman et al., 2019). The rising prevalence of BD among college students may be due, in part, to the fact that attending college generally entails moving away from home. This provides freedom from parental supervision, especially at a time when youth are likely to be experimenting and exploring various risky activities (Mosel, 2023). Furthermore, excessive alcohol consumption is seen as a vital part of a student’s identity, particularly among student athletes and college campuses provide numerous possibilities for BD (NIAAA, 2023). Despite improvements in college drinking interventions, alcohol consumption among college students remains high and students believe that alcohol use is not an issue (Patrick et al., 2023).

Despite numerous treatments aimed at lowering BD among college students and its accompanying repercussions, BD remains high. Targeting preventive and intervention efforts at young adults may boost effectiveness and prevent both the short- and long-term consequences of BD. National and local efforts to prevent BD among young adults include drinking age reduction regulations and media campaigns. Many college campuses have also implemented alcohol prevention initiatives, but many of them fail to produce significant effects. According to Talmon (2019), those in ‘Generation Z’ who have significant access to digital devices see communication via this technology (SMS, online messaging, email and mobile phone apps) as normal, comfortable and necessary in social settings. College students and young people are more accustomed to communicating privately on a regular basis via mobile devices. Furthermore, therapies delivered through mobile devices and technology increase this population’s receptivity to new information and verbalization. Peer-led treatments are increasingly being used in colleges and universities worldwide to prevent BD or promote safe drinking (Eaton et al., 2018; Pueyo‐Garrigues et al., 2023). The review found limitations in having high attrition rates between baseline and follow-up in several of the trials. Another drawback was the inability to respond to SMS messages.

BD is a pervasive issue affecting young adults across the globe (Courtney and Polich, 2009). It transcends geographic boundaries and socioeconomic disparities, making it a matter of global concern. Understanding effective interventions is essential to mitigate these global health challenges. High-income countries often serve as trendsetters in various domains, including public health and intervention strategies. Research conducted in these countries can influence policies and interventions not only within their borders but also internationally (McGregor et al., 2014). By examining BD interventions in high-income countries, this study contributes to the global dialog on alcohol consumption and prevention, providing valuable insights for countries at all income levels. One of the significant contributions of this research is the potential for interventions to be transferred across different contexts. Effective strategies identified in high-income countries can serve as models for adaptation in low- and middle-income countries, provided they are culturally sensitive and contextually relevant (McGregor et al., 2014). This research can facilitate knowledge transfer and help bridge the gap between diverse regions. A systematic review allows for a comprehensive comparison of interventions across high-income countries. By identifying differences in effectiveness, cultural sensitivity and potential biases, this study sheds light on the nuances of addressing BD in various settings. Policymakers and researchers worldwide can benefit from the insights provided by such comparative analyses.

BD’s consequences extend far beyond individual behavior, affecting public health and well-being on a global scale (World Health Organization, 2024). Addressing this issue among college students and young adults is a shared concern for countries worldwide. Therefore, research that offers evidence-based solutions is crucial to mitigate the negative impact of BD on societies and healthcare systems. Researchers and policymakers from various countries can benefit from the comprehensive overview provided by this study. It contributes to the global research landscape by aggregating evidence and offering a structured assessment of BD interventions.

The purpose of this present review was to identify current peer-reviewed research studies that identified BD interventions among college students focusing on preventing or practicing responsible drinking to develop future recommendations. The review focuses on the study’s design, number of study participants, type of intervention and intervention description, key findings and limitations. It is envisaged that the review will add to the evidence-based literature and guide health practitioners and researchers about the viability and efficacy of therapies for reducing BD in young adults, particularly those in college.

## Methods

The inclusion criteria for the studies in this systematic review were as follows: (i) the article had to be published in English, (ii) it had to evaluate a BD reduction program among college students or young adults between the ages of 17 and 24 years, (iii) it had to be published between 2017 and 2023 and (iv) it had to be a randomized control trial (RCT). Exclusion criteria included research not published in English, systematic review articles, qualitative studies, designs other than RCTs and discussion articles on college students drinking with no findings. We considered multiple published studies on the same intervention together as one study.

The main outcomes measured the students’ health outcomes in terms of the beneficial effects of the intervention.

- Prevention of BD among college students.
- Reduction of BD among college students.
- Quitting BD among college students.
- Reduction in consequences associated with BD among college students.
- Practicing responsible drinking and/or abstinence.

### Search strategy

We used the Preferred Reporting Items for Systematic Reviews and Meta-Analysis (PRISMA) standards to conduct a search of various interventions. The authors identified and looked for relevant research in three databases: MEDLINE (PubMed), APA PsycINFO and ERIC. Boolean operators “AND” and “OR” were utilized for different combinations of keywords, substance abuse, BD, alcohol abuse, alcohol interventions and BD interventions.

### Search details: BBD AND interventions AND college students AND interventions filters: From 2017 to 2023.

(("binge drinking"[MeSH Terms] OR ("binge"[All Fields] AND “drinking”[All Fields]) OR “binge drinking”[All Fields]) AND ("intervention s"[All Fields] OR “interventions”[All Fields] OR “interventive”[All Fields] OR “methods”[MeSH Terms] OR “methods”[All Fields] OR “intervention”[All Fields] OR “interventional”[All Fields]) AND (("college"[All Fields] OR “college s”[All Fields] OR “colleges”[All Fields]) AND ("student s"[All Fields] OR “students”[MeSH Terms] OR “students”[All Fields] OR “student”[All Fields] OR “students s”[All Fields])) AND ("intervention s"[All Fields] OR “interventions”[All Fields] OR “interventive”[All Fields] OR “methods”[MeSH Terms] OR “methods”[All Fields] OR “intervention”[All Fields] OR “interventional”[All Fields])) AND (2017:2023[pdat])

### Selection of studies

PRISMA were used. The authors prescreened the electronic search using the keywords identified in the electronic search. The authors received complete texts of all potentially relevant studies and analyzed the full texts that were to be included in the research.

### Article screening and data collection

**Figure 1.**
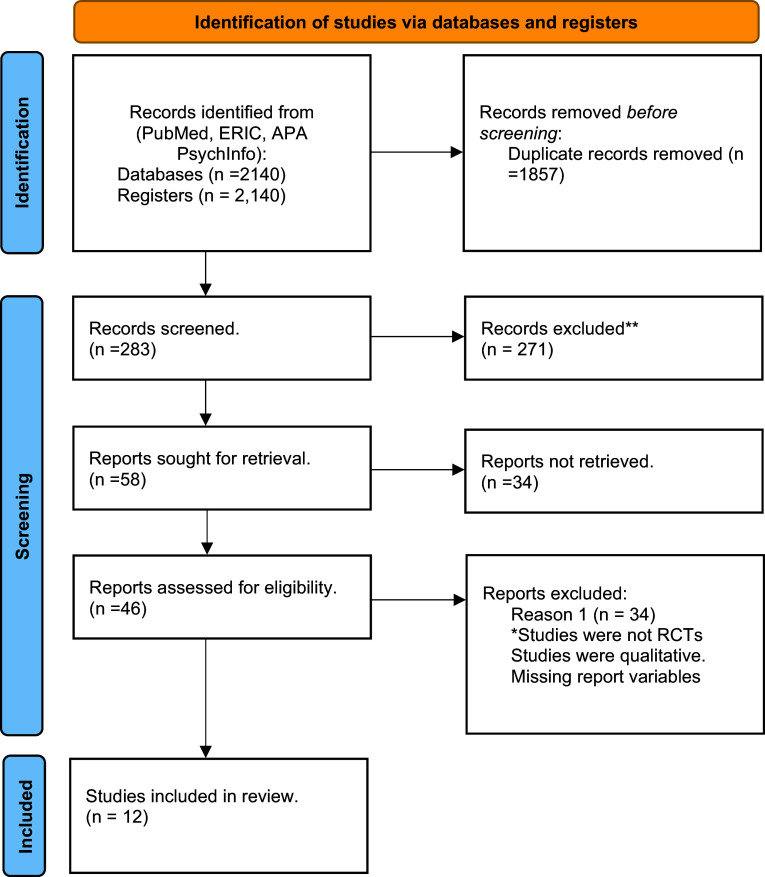
Search strategy using the PRISMA diagram.

After duplicates were removed, all studies were screened in two stages: titles/abstracts and complete texts. The abstracts and titles were examined in accordance with the inclusion criteria outlined above. If there was any ambiguity about the abstracts, the publications were included for full-text examination. One reviewing author independently re-reviewed all entire papers to confirm that they matched the inclusion criteria. If any disagreements emerge, they were handled by a second and third reviewing author. The researchers piloted and executed the data extraction procedure. The data were retrieved from the studies using a Microsoft Excel spreadsheet to obtain key information for the, following:

1. Last name of the author, year and country. 2. Population and sample size. 3. Study design. 4. Intervention and Description. 5. Salient findings. The reviewers verified the data extraction and a PRISMA flow diagram (please see Figure 1) and checklist (please see the Appendix) were prepared.

### Quality assessment

Study quality assessment or appraisal tools are served for the purpose of assessing the quality of research publications. Systematic reviews have included its use and have shown to be beneficial in evaluating the constraints of a research project. One aspect of critical analysis is the assessment of potential bias in research. The assessment of our studies’ risk of bias helped in assessing the internal validity of the included studies by the JBI’s critical appraisal tools. This entailed determining if the study was conducted without any systematic errors, thereby increasing the likelihood that the reported findings are accurate (Joanna Briggs Institute, 2023). The execution, analysis and interpretation of data in a research project may be susceptible to bias. Evaluating the potential for bias in research is a crucial component of doing a systematic assessment of quantitative data.

## Results

### Results of the search

After removing duplicates, the reviewers scoured various electronic databases and other resources specified in the methodology section and found 2,140 studies. For eligibility, 1,857 research studies were excluded based on the titles, theses, abstracts and full texts of 283 articles. Twelve articles were found to have met the inclusion criteria after reading these scholarly articles. The PRISMA flow diagram (Figure 1) describes the search approach in detail.

### Details of studies included

The reviewer has included trials in detail in characteristics of included studies in Table 1. Nine of the studies were conducted in the United States, two in the United Kingdom, and one (1) each in Spain and Italy. King et al. (2019), Lyden et al. (2022), Morris et al. (2022), Patrick et al. (2021), Suffoletto et al. (2019), Tahaney and Palfai (2017), Wisk et al. (2021) and Yurasek et al. (2017), included participants from the United States. Norman et al. (2017; 2019) included participants in the United Kingdom. Pueyo‐Garrigues et al. (2023) included participants from Spain and Fantini et al. (2023) included participants from Italy.

**Table 1.** Description of study populations in alphabetical order of first author (n = 12)

Author, year	Country	Sample population	Study design
Fantini et al., 2023	Italy	N = 144	Randomized control trial
King et al., 2019	USA	N = 51 n(f) = 60.8%	Randomized control trial
Lyden et al., 2022	USA	N = 891 Randomly assigned to assessment-only control group. n = 300, n = 531 is randomly assigned to the intervention group	Randomized control trial
Morris et al., 2022	USA	N = 73	Randomized control trial
Norman et al., 2017	UK	N = 2,951 s (experimental group) =2,682 Post-intervention measures s1(m) = 1,214, s2(f) = 1,444	Randomized control trial
Norman et al., 2019	UK	N = 407	Randomized control trial
Patrick et al., 2021	USA	N = 891 n (female) = 62.4% N (assessment-only) = 300 Stage 1 intervention = 295 Late stage 1 intervention = 296	Randomized control trial
Pueyo‐Garrigues et al., 2023	Spain	N = 50, Intervention group; n = 23, Comparison group; n = 27	Randomized control trial
Suffoletto et al., 2019	USA	N = 149 n (female) = 46% n (male) = 54%	Randomized control trial
Tahaney and Palfai, 2017	US & Canada	N = 113	Randomized control trial
Wisk, 2021	USA	N = 122	Randomized control trial
Yurasek et al., 2017	USA	N = 530; 96% Caucasian, 67% male BMI; Marijuana users n = 118	Randomized control trial

### Interventions

Details of the populations and interventions are described in Tables 1 and 2. Table 3 summaries the description of studies, sites (country), samplings and quality assessment of the studies. Included studies provided mobile-based/phone-based, or face-to-face, or computer-based interventions in the treatment. The shortest study was 2 weeks (Norman et al., 2019) and the longest study lasted 28 weeks (Suffoletto et al., 2019).

**Table 2.** Description of intervention programs, duration and outcomes in alphabetical order of first author (n = 12)

Author, year	Interventions/duration	Salient findings
Fantini et al., 2023	Duration: First intervention delivered within 30 days, second assessment from March to April (30 days) and last assessment after 6 months (September–October 2019). Well-being Intervention (WBI) and Lifestyle Intervention (LI); Six 2-hour sessions held every 10–15 days.	Regarding BD, well-being (WBI) and lifestyle interventions (LI) showed a very good outcome (group-by-time interaction was statistically significant for the total AUDIT-C score especially for WBI with *p* = 0.044) and LI with *p* = 0.016 in comparison to no intervention, from baseline to six-month follow-up
	Sessions delivered by a clinical psychologist. Brainstorming, role plays, group discussions and games, were used n. Lifestyle intervention was based on psychoeducation.	
King et al., 2019	Duration: Baseline (session 1 and 2) to 1-month follow-up.	BASICS significantly reduced BD and related consequences. Telehealth and face-to-face interventions can significantly reduce BD by *p*-value = .02
	Brief Alcohol Screening Intervention for College Students (BASICS) through telehealth compared to face-to-face	
Lyden et al., 2022	Duration: 3 months, The M-bridge study consisted of an embedded tailoring variable (self-monitoring their heavy drinking) which triggered the attempt to bridge students to the intervention; online chat with a health coach or receive a resource email.	The use of API rules reduced BD frequency by about 1 occasion per 2.5 months 95% CI: decrease of 1.45–0.28 occasions, with a *p*-value =0.005.
	The process connected students to the intervention by providing them with the option to engage in an online conversation with a health coach or get a resource email.	
Morris et al., 2022	Duration: Sessions 1–4 (4 weeks). Brief motivational intervention (BMI), including the paradigms and constructs for motivational information enhancement and cognitive-behavioral strategies.	Confidence to change; *p* < 0.001 Readiness to change, *p* = 0.002 Importance to change; *p* = 0.43 decrease in alcohol use; *p* = 0.01.
Norman et al., 2017	Duration: 3 weeks before starting college; 1 week, 1 month, and 6 months after entering college with follow-up link questionnaires that assessed their alcohol intake. Repeated TPB after 1 and 6 months.	TPB had a direct effect on BD at 6 months; *p* = 0.004. Reduction to non-significance when TBP values were controlled; *p* = 0.37.
	Theory of planned behavior; self-affirmation, a person’s implementation intentions; self-affirmation task (questionnaire about important values and attributes) and then finished the theory of planned behavior cognitions measures concerning BD. Before starting college, students were to complete measures of alcohol consumption.	
Norman et al., 2019	Duration: 2 weeks. Theory of planned behavior and Implementation intentions; After reading a summary of the health risks associated with BD.	Participants who formed an implementation intention engaged in BD less frequently at follow-up than participants who were not asked to form an implementation intention. After the intervention, participants engaged in BD reduced their drinking attitude (*p* < 0.01)
	The participants were to complete a self-affirmation log for the exercise implementation intention risk or not an exercise. Participants were then to complete full questionnaires regarding messages.	
Patrick et al., 2021	Duration: 30 days for self-monitoring strategies 60 days to transition from self-monitoring to bridging strategy first follow-up survey in December 2019, 2nd follow-up in April/May 2019. M-bridge; there were 2 stages. The first stage combined Personalized Normative Feedback (PNF) and self-monitoring.	Change in BD frequency from baseline to follow-up, *p* = 0.243.
	Participants were invited via email and text messages strategies included virtual chat with a health coach.	
Pueyo‐Garrigues et al., 2023	Duration: 50-min face-to-face motivational interview, conducted between October 2019 and April 2020. Peer-led BASICS session, the intervention group received a peer-led BASICS session that consisted of a one-off 50-min face-to-face motivational interview. Participants are provided with a personalized geographical feedback sheet, with topics such as participants drinking patterns, level of intoxication, perceived or actual drinking norms, alcohol expectancies, and individual risk factors.	The intervention group had a significant effect on BD episodes (mean = 0.3; *p*-value = 0.023). Participants in the intervention group reported significantly less BD and lower BAC than those in the control group (peak BAC, d = 0.98; BD episodes, d = 0.96).
Suffoletto et al., 2019	Duration: 12 weeks and 28 weeks. 5 Text message interventions; on days those participants drank (1–3 days per week); later they received ecological momentary assessments (EMA) of drinking plans and desire to get drunk. They were asked to report the number of drinks consumed the previous day.	Binge drinking reduced from 93% to 65% by week 12. Using all the text message interventions showed a significant reduction in binge drinking and heavy episodic drinking over time.
Tahaney and Palfai, 2017	Duration: One text a day for 30 days. Text messaging and web-based intervention: eCHECKUP TO GO-alcohol. Motivational techniques to provide individualized feedback were used.	Results showed that participants in the web-based intervention and text messages group showed less weekend BD. Individuals in the text messaging group did not exhibit significantly fewer alcohol-related consequences at follow-up (*p* = 0.33)
Wisk et al., 2021	Duration: 7.5-min of video content of intervention; 2-week follow-up. Social cognitive theory; Psycho-educational intervention targeting alcohol-related knowledge, attitudes and behavior of college students with type 1 diabetes.	The intervention model was highly acceptable and highly effective in reducing BD at follow-up. Participants reported a significant decrease BD 2 weeks after intervention (odds ratio = 0.48; 95% CL 0.31–0.75) compared to 2 weeks before the intervention (43/122, 35.2%)
Yurasek et al., 2017	Duration: 15-min Brief Advice; Online assessment 6 weeks after the BA; Follow-up assessment at 3, 6- and 9-months post-intervention. Brief assessment (BA), BAC, brief motivational intervention (BMI); Participants who reported binge drinking following BA sessions were randomized to a brief motivational intervention. Manualized BA (psychoeducation) was administered by a peer counselor.	Heavy-drinking and marijuana users may benefit from alcohol use interventions. Multiple regression models showed that baseline marijuana user status was not associated with changes in HED frequency or alcohol consequences following the interventions all with a *p*-value >0.05

**Table 3.** Description of studies, sites, sampling and quality assessment (n = 12)

No.	Author, year	Country	Sample population	Study design	Bias assessment criteria
1	Fantini et al., 2023	Italy	N = 144	Randomized control trial	11/13
2	King et al., 2019	USA	N = 51 n(f) = 60.8%	Randomized control trial	9/13
3	Lyden et al., 2022	USA	N = 891, randomly assigned to assessment-only control group; n = 300, n = 531 is randomly assigned to the intervention group	Randomized control trial	11/13
4	Morris et al., 2022	USA	N = 73	Randomized control trial	7/13
5	Norman et al., 2017	UK	N = 2,951 s (experimental group) =2,682 post-intervention measures s1(m) = 1,214, s2(f) = 1,444	Randomized control trial	11/13
6	Norman et al., 2019	UK	N = 407	Randomized Control trial	11/13
7	Patrick et al., 2021	USA	N = 891 N (assessment-only) = 300 Stage 1 intervention = 295 Late stage 1 intervention = 296	Randomized control trial	11/13
8	Pueyo‐Garrigues et al., 2023	Spain	N = 50, intervention group; n = 23, comparison group; n = 27	Randomized control trial	11/13
9	Sebire et al., 2019	USA	N = 149	Randomized control trial	11/13
10	Tahaney and Palfai, 2017	US & Canada	N = 113	Randomized control trial	8/13
11	Wisk et al., 2021	USA	N = 122	Randomized control trial	11/13
12	Yurasek et al., 2017	USA	N = 530; 96% Caucasian, 67% male BMI; Marijuana users n = 118	Randomized control trial	11/13

## Discussion

The aim of this study was to conduct a systematic assessment of scholarly literature on interventions aimed at lowering or reducing BD or practicing responsible drinking among college students and students of college-going age. The study designs in the articles were comparable as they were all RCTs. Despite the fact that numerous RCTs were employed in this literature, more are required in this domain. This is suggested since RCTs are considered the gold standard for program evaluations. More studies in these areas will allow researchers to undertake larger meta-analyses, sensitivity and subgroup analyses to provide common effect estimates for measures such as alcohol-use frequency and alcohol-related outcomes.

Few interventions (n = 5) employed behavioral theory, specifically, social cognitive theory and theory of planned behavior. One article combined the theory of planned behavior, self-affirmation and implementation intentions. In terms of the intervention approach, two studies used M-bridge (n = 2). M-bridge intervention was conducted among freshmen. The aim of the M-bridge was to develop an adaptive preventive intervention. Two articles used text messages and computer-based interventions. Two articles used brief motivational interventions (n = 2). Two articles used Brief Alcohol Screening and Intervention of College Students (n = 2). The final article used a well-being intervention (n = 1). The application of behavioral theories provides a good foundation for the implementation and study of the efficacy of an intervention in reducing BD or responsible drinking among college students. However, the theories used were not directed at behavior change but at behavior acquisition. There is a need to use behavior change theories such as the transtheoretical model (Han et al., 2017; de Freitas et al., 2020) or the multi-theory model (MTM) of health behavior change (Sharma et al., 2022). The use of such theories will enhance the efficacy and effectiveness of binge drinking interventions among youth. According to previous reviews and meta-analyses, screening and therapies in primary care settings are successful in lowering BD for up to a year (Bridges and Sharma, 2015). Hence, more long-term follow-ups are required by future researchers.

Then, 10 (Norman et al., 2017, 2019; Tahaney and Palfai, 2017; King et al. 2019; Suffoletto et al., 2019; Patrick et al., 2021; Wisk et al. 2021; Lyden et al., 2022; Fantini et al. 2023; Pueyo‐Garrigues et al., 2023) of the 12 interventions studied were shown to be successful in reducing BD among college students although the effect sizes were small to medium. One of the remaining interventions was not significant in lowering BD, but it was significant in lowering heavy marijuana users’ alcohol intake (Yurasek et al., 2017). The theoretical frameworks that produced significant changes were the theory of planned behavior, social cognitive theory, self-affirmation and implementation intentions. As suggested earlier, the use of fourth-generation MTMs can be used to enhance the effect sizes of future interventions (Sharma et al., 2022).

Nearly all the interventions in this review were delivered over the phone or through a computer. Some of the research studies were conducted in person or on college campuses. The findings of this systematic analysis indicate that treatments for lowering BD among college students can be carried out in a variety of media, particularly via mobile devices and emails. Future researchers must utilize technology to augment their interventions directed at youth to curtail BD.

The duration of the interventions ranged from 2 weeks to 6 months in this review. Most of the interventions were brief interventions (n = 4 weeks). Some of the interventions lasted for only 7.5 min of video content from peer counselors, psychologists and social workers. This form of intervention is a brief intervention. TPB, self-affirmation and social cognitive theories ranged from 2 to 4 weeks and were followed up after 6 months. Two of the interventions lasted for 6 months. There is a need for future researchers to follow-up the interventions over longer periods of time.

In terms of intervention fidelity rates or satisfaction, none of the studies reported employing process evaluation techniques to analyze program strategy execution. Process evaluations are becoming more common in pragmatic RCT and intervention trial for healthcare treatment and behavioral change modalities (French et al., 2020). These evaluations play a critical role in improving the knowledge, attitude and practice for change after the results of interventions. There has been little discussion on process evaluation in settings pertaining to college and university students. The concurrent process evaluation has also been utilized in peer-led intervention mechanisms where social media influencers can act as change agents (Sebire et al., 2019). Another example was a teacher-facilitated high-intensity interval training intervention to assess feasibility and efficacy in older adolescents (Harris et al., 2021). Another relevant example would be community health worker-delivered support intervention for children and adolescents living with HIV and their caregivers for fidelity, feasibility and acceptability of community-based intervention (Dziva Chikwari et al., 2018). In our review, while the majority of the studies used three measurements: pretest, posttest and follow-up, they did not employ process evaluations. Hence, future researchers testing efficacy must utilize process evaluation, especially for fidelity assessment and satisfaction.

### Implications for practice

BD interventions are more efficacious if newer behavioral theory models, such as the MTM of health behavior change, can be used by future researchers. The MTM has been utilized in a cross-sectional study to explain the change of BD to responsible drinking and abstinence behavior among college students (Sharma et al., 2018). The researchers concentrated on two concepts: initiation and sustenance. For initiation or starting the behavior change of transitioning from binge drinking to responsible drinking/abstinence, three constructs were operationalized. The first construct, participatory dialog, entailed underscoring advantages over disadvantages, the second construct, changes in the physical environment, included removing or reducing alcohol exposure in the individual’s surroundings, and the third construct, behavioral confidence, built the surety for change. To sustain the behavior change of switching BD to responsible drinking/abstinence, the following are needed: (i) emotional transformation whereby using emotions to develop goals for responsible drinking or quitting BD, (ii) practice for change or regular thoughts on the necessity of responsible drinking or quitting BD and ultimately (iii) changes in the social environment whereby obtaining family and friend support and assistance in maintaining the quitting behavior. This theory can be applied in developing future interventions for youth to quit BD.

### Limitations of the studies

The studies utilized in this research were composed of a small number of relevant research studies. The small sample sizes used in these studies were a constraint. Furthermore, the duration of intervention delivery and the follow-up period of the studies were short. Future researchers must work with larger sample sizes and follow-up with youth for longer periods of time to gauge the sustenance of behavior change. Furthermore, the majority of the studies did not undertake process evaluations. The inability to fully comprehend how and why particular approaches were successful or unsuccessful restricts the ability to create more specialized and focused interventions. The fact that the studies represented are western, industrialized nations, these findings may not be generalized to dissimilar populations. The study’s focus was on individuals between the ages of 17 and 24 years. Even though this is a crucial age range for binge drinking, it leaves out some young adults and older college students who may also engage in harmful drinking habits. Again, without teasing out the aforementioned demographic specifiers, we are unsure as to which subsets of college-aged students these findings may really apply to.

### Limitations of the review

First, publication bias may have played a role whereby studies with favorable results may have had a greater chance of publication and those with non-significant results have less chance. Second, this review included only papers published in English, eliminating those published in other languages that may have met the inclusion criteria. Third, systematic reviews of RCTs for interventional studies are widely recognized as the most reliable and rigorous kind of evidence. Despite the growing number of RCTs published in the field, our systematic review did not analyze other designs routinely used in interventions or management regimens for binge drinking behaviors in college and university students. The exclusion of qualitative studies may have limited our knowledge of the underlying causes and motives for college students’ binge drinking. Quantitative data can be complemented with qualitative information. Fourth, articles published from 2017 to 2023 were included in the study. This little window of time may have eliminated pertinent interventions that were carried out prior to 2017 or are still in progress after 2023. Finally, our systematic review was limited to the extraction of reporting selective outcomes. Only those indicators were extracted with selectively existent behaviors that were considered statistically significant for the results that aligned with research questions and interests. Future researchers must keep these aspects in mind when conducting further reviews.

## Recommendations

This systematic review would be beneficial to anyone working in the domains of public health, health education, college health and other allied health fields who work with substance and drug education. While this review demonstrates various efficacious interventions for reducing BD among college students, more research in this area is required, including adapting, utilizing and evaluating the effectiveness of various intervention approaches such as, but not limited to, BASICs, mHealth, self-affirmation, Mbridge and social cognitive theory, as well as process evaluation of program studies. Again, to avoid publication bias, researchers should also attempt to obtain unpublished data and gray literature and conduct a quality assessment analysis. Assessment and reporting on the heterogeneity of the included studies, both clinical and methodological, and exploration of potential sources of variations are crucial for future researchers.

As binge drinking is still a problem for those above the age of 17–24, future studies should account for extending the age range to include older college students and young adults. The inclusion of qualitative research can lead to a deeper comprehension of the social and psychological causes of binge drinking, enabling more thorough interventions. To evaluate the durability of behavior change over time, researchers should undertake treatments with extended follow-up times. This can be used to assess the sustainability of the reported declines in binge drinking. When developing interventions, researchers should consider adopting cutting-edge theories of behavior change, such as the MTM of health behavior change. These theories might offer a more thorough framework for comprehending and modifying binge drinking habits. Moreover, future research should also examine cross-cultural studies because they can shed light on how cultural variations affect binge drinking behaviors and guide culturally appropriate interventions. Finally, we encourage the development of comprehensive and multifaceted solutions for preventing binge drinking that consider both individualized and environmental aspects by working together with researchers, academic institutions and local communities. Future research can lead to more effective and specialized interventions aimed at lowering binge drinking among college students and young adults by addressing these constraints and putting these recommendations into practice.

## Conclusion

This systematic review supports the growing evidence that health interventions are a means of addressing binge drinking and warrants further development and study. The quality of evidence from the fewer studies supports the need for more research in this area. Despite having modest to moderate effect sizes, most of the interventions were helpful in lowering binge drinking. Despite this, there is room for advancement in the planning and execution of such initiatives. This study made several important recommendations, including the need to extend the age range of participants, include qualitative research, use longer follow-up periods, incorporate behavior change theories, carry out process assessments and use technology for intervention delivery. It is undeniable that binge drinking among young adults and college students continues to be a serious public health issue with negative effects across many domains. Although there has been progress in designing interventions, there is still more to be done to increase their sustainability and efficacy. We recommend policymakers, clinicians and educators design many interventions that will encourage college students or young people of college age to quit engaging in responsible drinking. More interventions based on behavior change sustenance are desperately needed in this field. Continued empirical research is required to determine the efficacy of strategies for reducing BD among college students on college and university campuses. We recommend policymakers, clinicians and educators design many interventions that will encourage college students or young people of college age to quit or adopt responsible drinking. More interventions based on behavior change sustenance are desperately needed in this field. Continued empirical research is required to determine the efficacy of strategies for reducing BD among college students on college and university campuses.

## Data Availability

Available from the corresponding author upon request.
