# Supplementary material for: A systematic review of binge drinking interventions and bias assessment among college students and young adults in high-income countries
Source: Glob Ment Health (Camb). 2024 Feb 23;11:e33. doi: 10.1017/gmh.2024.24 (PMC10988166; doi:10.1017/gmh.2024.24)
Supplement: Peer review history

# Editor report: Recommendation: A systematic review of binge drinking interventions and bias assessment among college students and young adults in high-income countries — R0/PR2

**DOI:** 10.1017/gmh.2024.24.pr2

**Affiliations:** Public Health, Section of Global Health, University of Copenhagen, Denmark

Please clarify the ‘global’ relevance of this research in the Introduction and throughout the manuscript. Please also make it clear in the title. Is this binge-drinking among college students in the U.S., in high income countries, globally, etc.?

# Editor report: Decision: A systematic review of binge drinking interventions and bias assessment among college students and young adults in high-income countries — R0/PR3

**DOI:** 10.1017/gmh.2024.24.pr3

**Affiliations:** Johns Hopkins University Bloomberg School of Public Health, United States

No accompanying comment.

# Editor report: Recommendation: A systematic review of binge drinking interventions and bias assessment among college students and young adults in high-income countries — R1/PR5

**DOI:** 10.1017/gmh.2024.24.pr5

**Affiliations:** Public Health, Section of Global Health, University of Copenhagen, Denmark

No accompanying comment.

# Editor report: Decision: A systematic review of binge drinking interventions and bias assessment among college students and young adults in high-income countries — R1/PR6

**DOI:** 10.1017/gmh.2024.24.pr6

**Affiliations:** Johns Hopkins University Bloomberg School of Public Health, United States

No accompanying comment.
